# Vacuum template synthesis of multifunctional nanotubes with tailored nanostructured walls

**DOI:** 10.1038/srep20637

**Published:** 2016-02-10

**Authors:** A. Nicolas Filippin, Manuel Macias-Montero, Zineb Saghi, Jesús Idígoras, Pierre Burdet, Angel Barranco, Paul Midgley, Juan A. Anta, Ana Borras

**Affiliations:** 1 Nanotechnology on Surfaces Laboratory, ICMS Materials Science Institute of Seville (CSIC-US). C/Americo Vespucio 49, 41092, Seville (Spain); 2 Department of Materials Science and Metallurgy, University of Cambridge, 27 Charles Babbage Road, CB3 0FS, Cambridge (United Kingdom); 3 Departamento de Sistemas Físicos, Químicos y Naturales Universidad Pablo de Olavide, Carretera de Utrera km1, 41013 Seville (Spain)

## Abstract

A three-step vacuum procedure for the fabrication of vertical TiO_2_ and ZnO nanotubes with three dimensional walls is presented. The method combines physical vapor deposition of small-molecules, plasma enhanced chemical vapor deposition of inorganic functional thin films and layers and a post-annealing process in vacuum in order to remove the organic template. As a result, an ample variety of inorganic nanotubes are made with tunable length, hole dimensions and shapes and tailored wall composition, microstructure, porosity and structure. The fabrication of multishell nanotubes combining different semiconducting oxides and metal nanoparticles is as well explored. This method provides a feasible and reproducible route for the fabrication of high density arrays of vertically alligned nanotubes on processable substrates. The emptying mechanism and microstructure of the nanotubes have been elucidated through SEM, STEM, HAADF-STEM tomography and energy dispersive X-ray spectroscopy. In this article, as a proof of concept, it is presented the straightforward integration of ZnO nanotubes as photoanode in a photovoltaic cell and as a photonic oxygen gas sensor.

Inorganic nanotubes (NTs) present a viable alternative to the nowadays widely used carbon NTs since they enable to exploit material-specific properties, allowing the development of biomedical, photochemical, electrical, and environmental applications^1^,^2^,^3^,^4^. Concretely, ZnO and TiO_2_ NTs have been developed due to their suitable electrochemical properties, excellent solution stability, and relatively low toxicity^1^,^2^,^3^,^4^,^5^,^6^,^7^ with enhanced performance in applications as photocatalysis, solar cells and nanogenerators, sensoring, optical devices, antifogging, self-cleaning, smart-surface and biomedical coatings^7^,^8^,^9^,^10^,^11^,^12^,^13^,^14^,^15^. In a general way, the methods used for the fabrication of nanotubes can be divided into four wide groups^3^,^16^,^17^,^18^,^19^: formation of NTs due to morphological constrictions by vapor-liquid-solid (VLS), vapor-solid (VS) and other catalytic methods^16^; electrospinning^18^; anodization^1^,^19^ and treatment of solid nanofibres (NFs) and nanowires (NWs) in order to remove the inner part^20^,^21^ and, finally, the use of templates^22^,^23^,^24^,^25^,^26^. There are two principal approaches in the last group, namely the use of anodized alumina as hollow 1D template that can be filled through solution-based or vacuum methodologies^23^,^24^ and the application of the atomic layer deposition (ALD)^25^,^26^ of inorganic precursors using as substrate pre-grown 1D nanostructures and fibers. The methodology presented herein might be included in the last group of methods, with three important particularities. Firstly, the nanomaterials used as template are supported single crystalline organic nanowires (ONWs) fabricated by physical vapor deposition (PVD) of small-molecules^27^,^28^,^29^,^30^,^31^; secondly, the metal oxide layers forming the walls of the NTs are prepared by remote plasma assisted fabrication^32^,^33^,^34^ and finally, the template is easily removed by annealing of the ONWs at mild temperature. These characteristics render a full vacuum approach for the fabrication of NTs with tuneable length, hole dimensions and shapes, tailored wall composition, microstructure, porosity and structure. Thus, we denominate these nanotubes as three dimensional (3D) aiming to stress that the nanotubes are form by tailored walls with controlled nanostructure and thickness (from a few nanometers up to several hundreds). As far as we know, there is not reported any methodology with similar outcomes. It is also worthy to stress the generality of the method developed from different points:

- Shell composition and microstructure: Although we focus herein in the growth of two key wide band gap semiconductors (ZnO and TiO_2_), the formation of the inorganic shell is straightforward applicable to other materials available by vacuum deposition at low and mild temperatures. In fact, results on the formation metal nanoparticles are also included as an example. In addition, the fabrication of multishell nanotubes is easily achievable by sequential deposition of metal or metal oxide materials. The microstructure and properties of the shell are straightforwardly tuneable by controlling experimental conditions such as plasma gas composition and pressure, substrate temperature, etc. The thickness of the shell depends on the growth rate and time with rates highly competitive in comparison with other established techniques such ALD. As a matter of the fact, we have developed 3D nanotubes with walls presenting microporous (nano-TiO_2_), columnar mesoporous (meso-TiO_2_) and wurzite ZnO characteristics and the multishell formed by sequential formation of these materials but the protocol is easily extendable to other metal and metal oxides deposited by PECVD, DC or magnetron sputtering^35^,^36^,^37^,^38^,^39^,^40^.
- Substrate: Main protocols involved in the template formation of the 3D nanotubes are fully compatible with the use of silicon, optical substrates, metal oxide thin films, metal nanoparticles, and flexible substrates as PDMS, PET, etc. opening the way for a straightforward growth of these 1D nanostructures on electrodes, processable substrates and devices.
- Applications: The versatility of the method in terms of nanotubes composition and properties provides an unprecedented route for the fabrication of supported multifunctional nanotubes and single-wire devices.

## Results and Discussion

### Fabrication and characterization of 3D TiO_2_ and ZnO nanotubes

**Figure 1:**
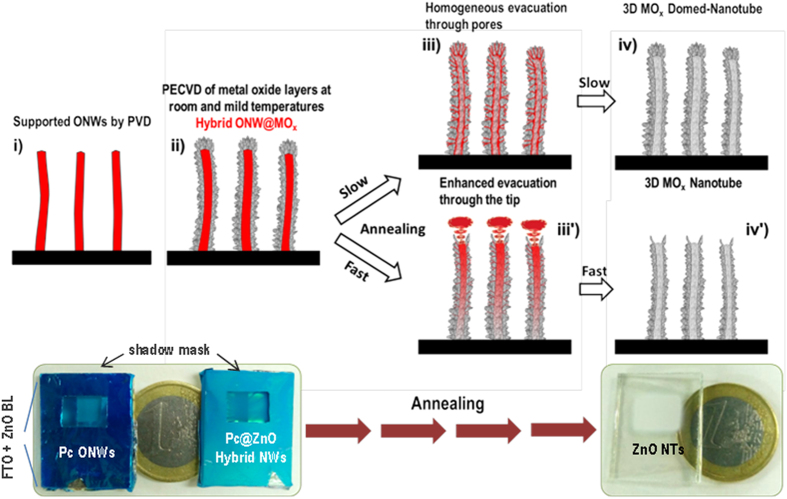
Representation of the steps involves in the formation of the oxide nanotubes.

**Figure 2:**
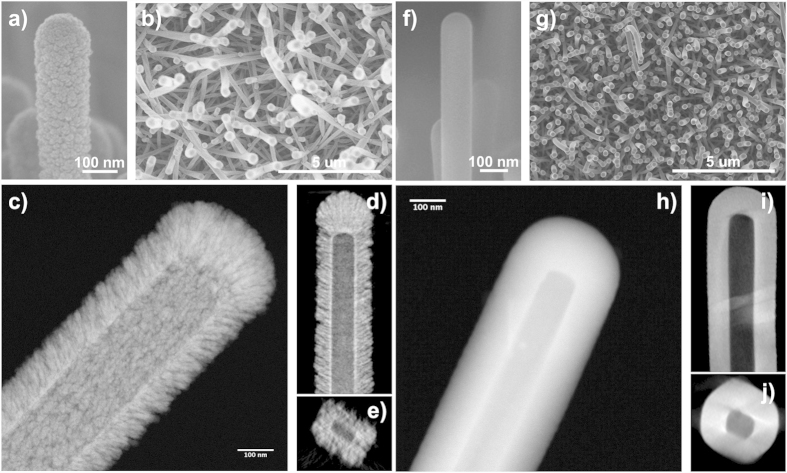
Meso- (**a–e**) and Nano (**f-j**) TiO_2_ 3D Nanotubes. SEM (**a,b**)/(**e,f**) and HAADF-STEM (**c–e**)/(**g–i**) characterization of nanotubes formed by columnar/continuous nanoporous TiO_2_ walls. Panel (**a**) shows the globular surface characteristic of the columnar TiO_2_ fabricated by PECVD at RT under oxygen plasma. HAADF-STEM image in (**c**) demonstrates the mesoporous microstructure of the wall with columns growing radially from the evacuated core. (**d,e**) are orthoslices through the 3D HAADF-STEM reconstruction were the rectangular cross section of the tube is clearly appreciable (**e**) along with a continuous interface between the empty core and the TiO_2_ columns. Panel (**h**) presents the smooth surface characteristic of this TiO_2_ deposited by PECVD at room temperature under argon/oxygen plasma. The wall is a continuous layer showing no visible porous under inspection by HAADF-STEM (**h**). Orthoslices through the 3D HAADF-STEM reconstruction show the squared cross section of the core of the nanotube (**i-j**) and the homogeneous thickness of the TiO_2_ wall.

**Figure 3:**
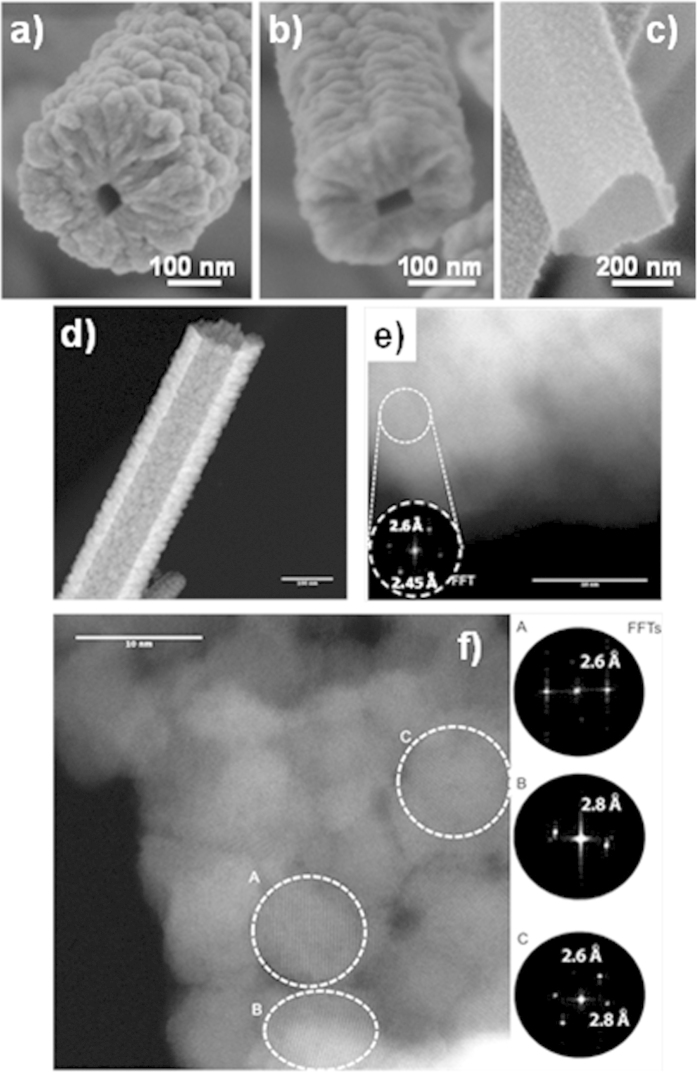
ZnO 3D Nanotubes. (**a–c**) SEM characterization of different 3D nanotubes formed by ZnO showing details of the columnar microstructure and different shape and lateral sizes of the cavity in open nanotubes. (**d**) STEM micrograph of a ZnO nanotube, (**e,f**) HR-STEM micrographs of a ZnO nanotube where the outer and inner wall are correspondingly exposed and analyzed. The insets show the FFTs of the marked areas in the images.

Figure 1 shows the different steps for the fabrication of the 3D nanotubes along with photographs of real samples for each step. Formation of the ONWs template (Fig. 1i) is carrying out using a temperature controlled PVD process that allows the growth of squared NWs and nanobelts. The organic nanowires are formed by self-assembly of small molecules (porphyrins, phthalocyanines and perylenes) on substrates of different chemical nature, including metal nanoparticles, polymers and oxide thin films^30^,^31^,^32^,^33^,^34^. Looking to the final applications of the nanotubes, we have deposited ONWs on commercial FTO substrates, ZnO thin films deposited by PECVD on FTO for the fabrication of the DSCs and SiO_2_ nanocolumnar substrates fabricated by glancing angle vacuum deposition (GLAD)^31^,^36^,^37^ for the photonic sensor. The second step (Fig. 1ii) represents the conformal deposition by PECVD of the metal oxide shell on the as-grown ONWs. Critical advantages of the use of PECVD for the formation of such hybrid NWs are the vertical alignment of the final nanostructures and the formation of the inorganic shell with no damage of the organic structure in the core. Additional details on the fabrication of the hybrid core@shell nanowires can be found elsewhere^32^. Two different TiO_2_ shells were prepared, labelled as meso and nano to distinguish their characteristic porosity, columnar and mesoporous in the first case and continuous and microporous in the second one^34^. The prepared ZnO showed a globular-columnar microstructure in the wurzite phase. The third step represents a leap forward in our previous development and consists of annealing the hybrid nanowires under vacuum conditions (Fig. 1iii,iii’)) in order to evacuate the organic template and, finally, the last step (Fig. 1iv,iv’) is the cooling down of the samples in vacuum, avoiding water condensation in the highly porous nanotube walls. Figures 2 and 3 summarize representative FESEM and HAADF-STEM results obtained after the post-annealing of MePTCDI@TiO_2_(meso) (Fig. 2), MePTCDI@TiO_2_(nano) (Fig. 2), and MePTCDI@ZnO (Fig. 3) nanowires. It is interesting to note that by controlling the annealing parameters, the nanotubes either keep the original domed shape with rounded tip (Figs 2 and 3), or they appear open on the top (Fig. 3a–d). Increments of the temperature slope lower than 10 °C min^−1^ lead to closed nanotubes meanwhile faster temperature scan rates result preferably in open nanotubes (see Steps iii) and iii’) in Fig. 1). In these figures it is possible to appreciate that the original morphology of the hybrid nanowires, including their preferential vertical orientation (Fig. 2b,f)), is preserved after the evacuation process. Moreover, the NTs remain supported on the substrate where the original cores, i.e. the ONWs, were grown with no evidence of deformation or collapse. This result is of special relevance since it settles the path for the integration of the 3D-NTs on processable substrates and devices.

Regarding the formation of tailored porous TiO_2_ shells, Fig. 2a shows how the NTs fabricated under the TiO_2_-mesoporous conditions (see Methods) depict a rough and globular surface in good concordance with a columnar microstructure radially distributed along the NW length (Fig. 2c,d). These columns present a diameter distribution between 5 and 20 nm with pores comprised in both the mesopore (2 < d < 50 nm columnar interdistance) and the micropore range (d < 2 nm inherent to the distribution of the material forming the columns). It is worth mentioning that the HAADF-STEM reconstruction in Fig. 2d) (see also Video 1 in the Supplementary Information (SI)) demonstrates the formation of a continuous interface between the columns and the empty core of the order of the tens of nanometers that is probably responsible for the good mechanical stability of the microstructure. Figure 2f-h) shows the homogeneous microstructure and smooth surface formed under the TiO_2_-nanoporous conditions (Fig. 2f). In this case, the shell presents a continuous cross section (Fig. 2h) with non-appreciable pores. In previous publications, we have demonstrated that these experimental conditions lead to the formation of microporous thin films with a relative high volume of pores^38^. On the other hand, Fig. 3 gathers representative SEM, HAADF-STEM and HR-STEM images of the ZnO shells. The shell is formed by globular-columnar features growing from a granular interface (Fig. 3a–d). PECVD ZnO thin films are crystalline even deposited at room temperature^33^. However, analyzing the XRD diagrams of supported ZnO nanotubes and comparing them with the reference ZnO thin film, i.e. the layer deposited under the same experimental conditions on a Si(100) substrate, there are some interesting differences (Fig. S1 in the SI). An important feature is the non-texturized character of the supported NTs as evidenced by the presence of an intense (002) peak along to the (100) and (101) in comparison with the XRD diagram corresponding to the thin film that is dominated by the (101) peak. It is also interesting to notice that the ZnO nanocrystals present some internal stress as demonstrated by a displacement to the left on the diffraction peaks. From previous studies regarding the growth of nanocrystalline plasma materials it is well known that thinner films present a higher level of stress^33^ and a low degree of texture development. The smaller thickness deposited on the 1D nanostructure is a consequence of the much larger effective area to be covered in this case. Scherrer equation applied to the diffraction peaks of the nanotubes sustains a crystallite size of ~21 nm. Figure 3(d) shows a low magnification HAADF-STEM image of a ZnO nanotube where it is possible to appreciate an inner hollow core surrounded by ZnO globular grains. Electrical conductivity of the ZnO nanotubes is a key property for many different applications, such as solar cells or piezoelectric devices and strongly depends on the porosity, crystallinity, grain boundary and crystal orientation of the nanostructured materials^7^,^39^. Figure 3e shows aberration corrected STEM images of the outer side of a nanotube wall formed by ZnO single crystalline columns. A fast Fourier transformation (FFT) of the image in the selected areas (see inset) reveals 2.6 and 2.45 Å which correspond to the typical distances between the (002) and (101) planes, respectively. Figure 3f presents a high resolution micrograph of the inner nanotube wall where it is possible to assess the porous size, crystal planes and grain boundaries. At first glance, the material shown in this figure is heavily packed with narrow spaces between grains. However, it is clearly visible the presence of open pores of about 2–3 nm width that are connecting the inner hollow with the exterior. We will discuss below how this porosity is crucial to evacuate the organic core and determinant in the electron transport properties of the NTs. The FFT analyses in regions A and B yield plane distances of 2.6 and 2.8 Å that correspond to the (002) and (100) planes respectively. The FFT of the C zone results in both distances supporting that the two planes are simultaneously observed. These results are in good agreement with the XRD diagrams in the Figure S1 of the SI where peaks corresponding to the planes (100), (002) and (101) are present. Several additional features of the developed methodology deserve to be stressed herein. In one hand, the thickness of the 3D nanotubes is easily controlled and defined by the deposition time during step ii). The SEM images in Fig. 3a–c show examples of open ZnO nanotubes with wall thicknesses of 200 nm, 80 nm and 20 nm respectively where the empty core is clearly visible. The wall thickness tends to be thicker on the top of the NT (Fig. 2d) because of the self-shadowing effect during the inorganic shell growth by plasma deposition. This effect is more pronounced for thick nanotubes and is directly linked to their vertical alignment^32^. Growth rate is relatively high with depositions as fast as 4 nm/min. The processes are carried out at low temperature and in remote deposition conditions, being compatible with temperature-sensible and delicate substrates. On the other hand, the created hollow replicates the shape of the organic nanowires, characterized by flat inner walls and squared (Figs 2i and 3a) or rectangular (Figs 2e and 3b) sections. It is also worth noting the flatness of the inner face (e.g. Fig. 2d,i). Thus, the outer parts present the typical surface roughness of the TiO_2_ or ZnO thin films meanwhile, the interface between the empty core and the shell keeps memory of the smooth molecular surface of the single-crystal wire template. The length of the NTs is as well easily tuneable by the deposition time applied in the formation of the ONWs (step i), ranging between the 500 nm and several tens of micrometers.

### Multishell 3D oxide nanotubes

**Figure 4:**
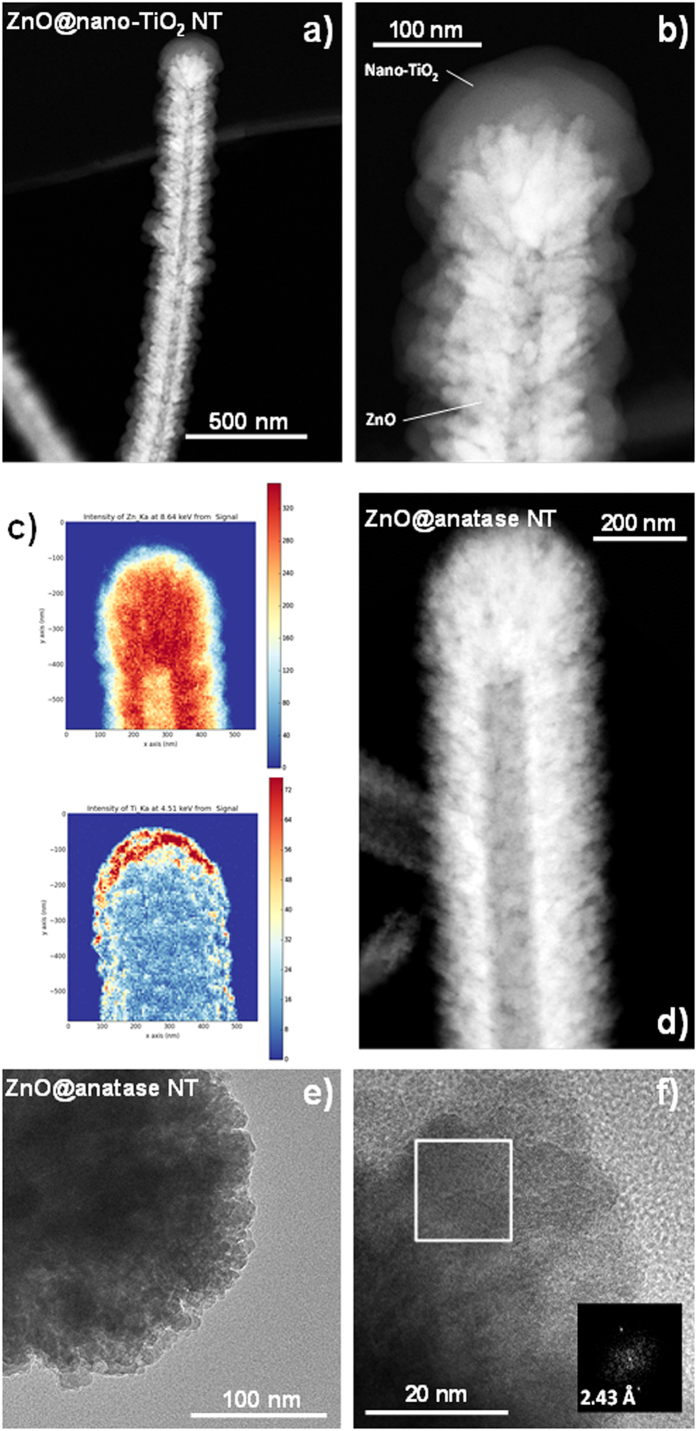
Multishell 3D Nanotubes. (**a**,**b**) HAADF-STEM micrographs of a ZnO@nano-TiO_2_ nanotube at two magnification scales showing the homogeneous coverage of TiO_2_ along the ZnO nanotube length (**a**) and a detail of this complex nanostructure (**b**). (**c**–**f**) Formation of an anatase layer on top of a ZnO nanotube. Distribution of Zn (up) and Ti (down) in the resulting EDX maps (**c**) obtained from the ZnO@anatase nanotube (**d**). Bright field TEM (**e**) and HRTEM (**f**), the inset showing the FFT of the selected area.

The PECVD technique allows the formation of multilayer systems of metal oxides by simply alternating the metalorganic precursors within the reactor without the need to expose the interfaces to air. We have applied this concept for the fabrication of multishell NTs, i.e. nanotubes with 3D walls formed by layers of different metal oxides (Fig. 4). Figure 4(a,b) shows the formation of a ZnO@nano-TiO_2_ multishell, where the crystalline ZnO layer was first deposited on the ONWs template and the nano-TiO_2_ subsequently fabricated on the ONW@ZnO system. After evacuation of the organic compound, the final nanostructure is defined as a ZnO@nano-TiO_2_ nanotube (see EDX analyses in Fig. S2). The HAADF-STEM image in Fig. 4a demonstrates the homogeneous deposition of the second layer on top of the ZnO shell. The microstructure of the TiO_2_ shell remains continuous and microporous (compare with Fig. 2). Moreover, it conformally follows the roughness of the ZnO surface (Fig. 4b). The method is also extendable to the fabrication of two crystalline shells. As an example, Fig. 4c–f) shows the formation of TiO_2_ anatase grains as external shell on the top of the wurzite ZnO layer. It is noticeable in the EDX maps (Fig. 4c) that even for such a low thickness (20 nm) there is anatase covering the whole ZnO shell. The FFT analysis of the selected area in Fig. 4f is in good agreement with the preferential formation of (004) planes as previously published for PECVD polycrystalline TiO_2_ thin films^40^. This methodology is straightforwardly applicable to other oxides deposited by PECVD like SiO_2_, SiO_x_C_y_H_z_, Al_2_O_3_, Ta_2_O_5_, oxynitrides, doped oxides, etc. and even to metal (Au, Ag) layers deposited by magnetron and DC-sputtering^35^,^36^,^37^,^38^,^39^,^40^. As example, Video 2 in the SI shows a 3D HAADF-STEM reconstruction of an Ag-NPs@ZnO@nano-TiO_2_ nanotube where silver nanoparticles (NPs) were deposited by DC sputtering in a previous step to the formation of the ZnO shell. We believe the virtually universal character of the developed procedure and the ample library of synthesized materials opens the way to the fabrication of multishell 1D nanostructures with potential applications as single-wire devices.

### Following the emptying mechanism of hybrid ONW@MO_x_ nanowires

**Figure 5:**
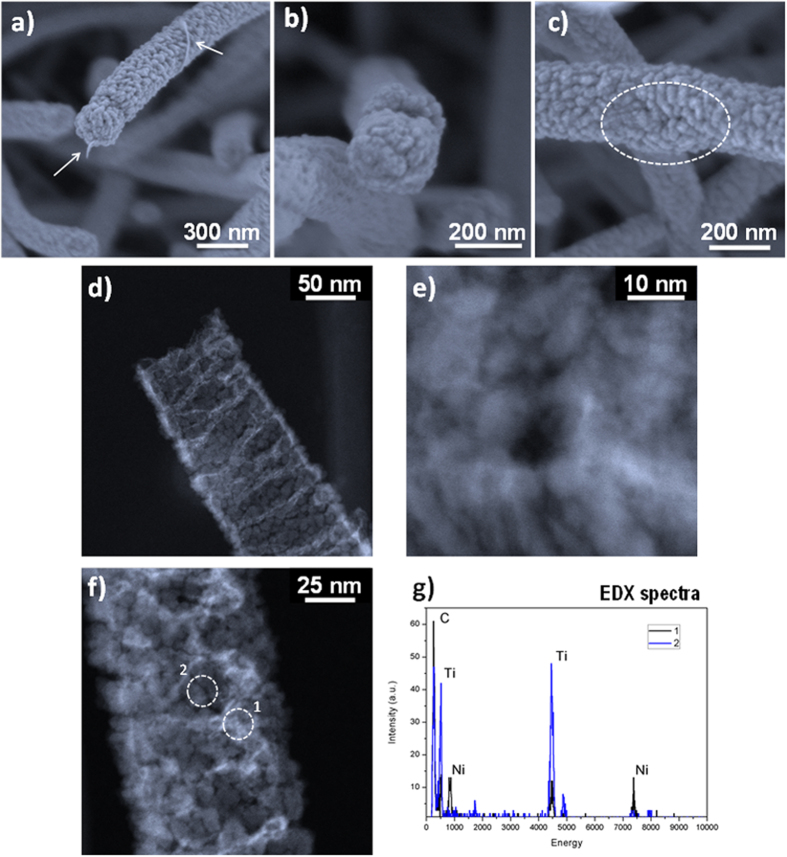
Characterization of the emptying process. (**a**–**c**) SEM micrographs of hybrid MePTCDI@ZnO after heating for 30 min in high vacuum, where the emptying process is clearly observable: arrows in (**a**) indicate the accumulation of the organic molecule; (**c**) shows one of the leaking points of the nanostructure. (**d**–**f**) HAADF-STEM micrographs of hybrid NiPc@TiO_2_ after a partially completed emptying process where the brighter regions correspond to the Ni in the NiPc molecule as it is corroborated by the EDX comparison in (**g**) between the areas marked as 1 and 2 in (**f**).

An important characteristic of the NTs’ microstructure is their tailored porosity. In the case of the meso-TiO_2_ and ZnO open pores connecting the inner channel with the exterior are present. Such porosity positively contributes to the evacuation of the organic template after annealing in vacuum. EDX (Fig. S3), XPS (data non-shown) and UV-Vis spectroscopy results (Fig. S4) support that after Step iv) the organic core is completely removed. Figure S4 compares the UV-Vis spectra of PtOEP@ZnO NWs with the corresponding ZnO nanotubes after the vacuum annealing at 280 °C during 30 and 60 minutes at 10^−6^ mbar. The spectrum of the as prepared samples shows a high absorption in the visible range. However, after the first 30 minutes of annealing, the UV-Vis spectrum changes, resulting in a considerable increment of transparency with yet residual organic adsorption bands (partially evacuated in Fig. S4). These bands disappear after 60 minutes of evacuation. In all the cases the spectra are significantly dominated by light scattering effects related with the size and distribution of the 1D supported nanostructures. Further information on the evacuation mechanism was obtained after characterization of different nanowires (Fig. 5) in intermediate stages of post-annealing, i.e. before the organic molecules were completely removed from the inorganic shell. Figure 5a–c gathers characteristic SEM micrographs of the emptying process. The organic compound starts to segregate on the surface of the nanotube in the form of thin stripes (Fig. 5a). Because of the columnar microstructure of the shells (ZnO and meso-TiO_2_) it is possible to observe how some porous channels weaken, provoking the detachment of small grains (e.g. Fig. 5c). Such effect is more important on the tips of the wires leading to tip detachment under appropriated annealing conditions as schematized in Fig. 1. To have access to the interior of the nanostructure during the emptying process, a HAADF-STEM analysis has been carried out for partially evacuated samples (Fig. 5d–f). Experiments were carried out with NiPc@meso-TiO_2_ as starting hybrid nanowire in order to enhance the contrast between the organic compound and the inorganic shell. It is possible to appreciate that the remaining organic material redistributes in the form of long and irregular stripes that lay close to the inner walls (brighter regions in Fig. 5d). Besides, the porous structure appears well defined, evidencing a pore size distribution of a few nanometers. Looking closer to these pores, Fig. 5e, it is possible to see halos of lower contrast surrounding them. We attribute these to the leftover organic material precipitated during the evacuation through the pores. To verify this, EDX analysis was performed on areas marked in Fig. 5f as 1 (brighter region) and 2 (darker region). The resulting spectra are compared in Fig. 5g showing that zone 2 has an important amount of titanium, while zone 1 presents nickel and carbon as main components. It is also important to remark that after completing the emptying process there is no trace of the small-molecules forming the original ONW template. Therefore, these results conclude that the organic compound is released through the connected porosity on the inorganic shells without apparent decomposing. In the cases analysed in Fig. 5, the final situation of the nanotubes is open but similar results were found for the domed nanotubes. Figure 1iii,iii’ intends to illustrate the intermediate steps. The increased pressure created in the interior of the inorganic shell by partial sublimation of the organic core would be released by diffusion of the organic part through the porous structure in a continuous and homogeneous way. This take place when the hybrid nanowires temperature is slowly incremented until the organic molecule sublimation temperature, which leaves the inorganic shell microstructure untouched (Fig. 1iii). For faster temperature scan rates the overpressure conditions inside the nanowire drive to the preferable detachment of the tip, likely due to its different grain or columnar orientation (Fig. 1iii’). The last route makes accessible the inner hollow of the nanotube as presented in Fig. 5a–d.

### 3D ZnO Nanotubes-Based Dye Sensitized Solar Cells

**Figure 6:**
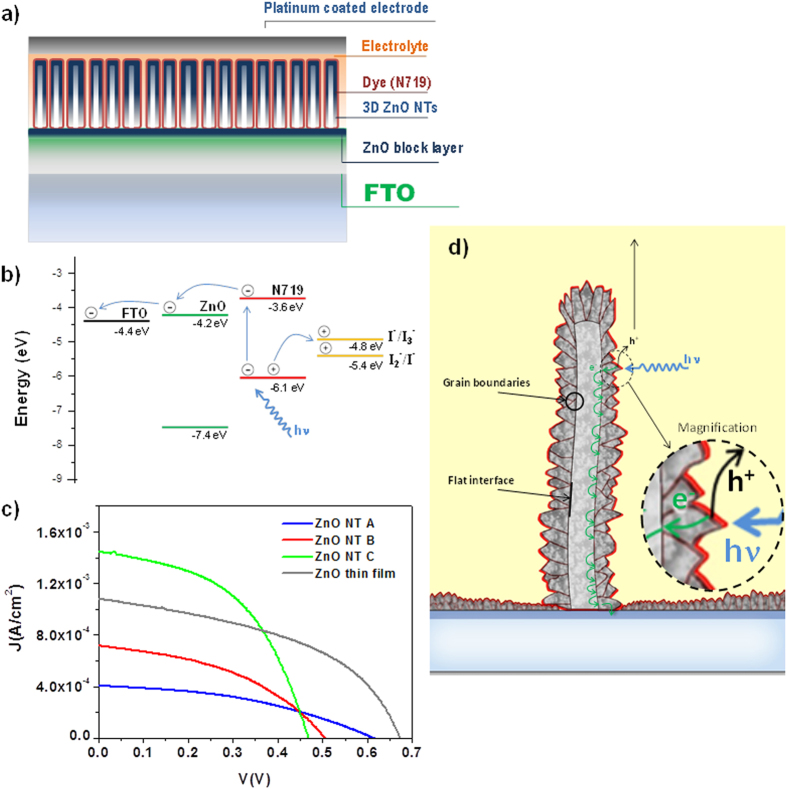
Schematics of the DSC device based on ZnO 3D NTs (**a**) and energy levels (**b**). (**c**) IV curves for the DSCs assembled with NTs of three wall thickness (A: 20 nm, B: 80 nm, and C: 250 nm) and a 800 nm thick ZnO porous thin film as a reference.; (**d**) proposed model for the solar cell conduction.

ZnO nanostructures in the form of thin film, nanowires, nanotubes and hierarchical nanostructures have been used as photoanodes in dye sensitized solar cells (DSC) as an alternative to TiO_2_ because their excellent transport properties^41^,^42^. In addition, the application of this semiconductor oxide to the emerging field of the perovskite solar cells has also been recently explored^43^. In fact, nanocolumnar ZnO thin films deposited by PECVD were reported last year as photoanode for both dye-sensitized^44^ and perovskite solar cells^45^. In the first article the electron-transport properties of the highly crystalline and texturized PECVD ZnO film were compared to that of a nanoparticulate layer by means of small-perturbation electrochemical techniques. In this way it was demonstrated that the electron transport in the case of the texturized film was determined by Fermi-level pining that made voltage independent, in contrast to the nanoparticulate film. On the other hand, a stimulating power conversion of 4.8% was obtained for the solid-state solar cell by depositing CH_3_NH_3_PbI_3_ on a porous ZnO nanocolumnar layer. Taking into account such promising antecedents, the main goal of this section is to demonstrate the suitability of the method for the direct implementation of the 3D ZnO NTs as photoanode in a DSC and to study their charge transfer and electron transport properties. For the first time the influence of NT wall-thickness on DSCs performance is introduced and analysed. The length of the NTs was kept constant owing to prior studies of its impact in DSCs^46^. It is important to note that no efforts have been carried out to optimize the efficiency, as this requires to look for the appropriate dye, sensitization procedure and choice of electrolyte, which is out of the scope of this study. To analyse the electron dynamics in the 3D NTs is especially interesting since its results are extendable to the performance of the 3D NTs in other devices such as the already mentioned perovskite solar cells, piezoelectric nanogenerators, gases and UV sensors. Three different types of samples have been chosen for this study with a similar NT density and length but different NT-wall thicknesses. Fig. S5 shows several SEM micrographs of the vertical aligned nanotubes utilized as photoanodes and Fig. 1 presents the photographs of the photoanodes previous to the assemble of the cell. The section of the nanotubes in each case is shown in the insets being the mean values for sample A: 20 nm, B: 80 nm and C: 250 nm. From now on, we will refer to the samples with these labels. Figures 6, S6 and Table 1 gather selected photovoltaic parameters for the solar cells fabricated by using the three photoelectrodes including a ~800 nm ZnO PECVD thin film electrode (Fig. S5) as reference to compare the performance of the nanotubes with respect to the nanocolumnar films. Figure 6a shows a schematic of the solar cell structure addressing each of the components: conductive transparent substrate (FTO), ZnO blocking layer, vertical aligned ZnO NTs, N719 dye and electrolyte with the energy levels diagram depicted in Fig. 6b 47. In Fig. 6c significant changes are observed in the IV curves as a function of the NT wall thickness. The first difference is that the open circuit voltage decreased for thicker NTs. This parameter depends on the recombination rate from the ZnO electrode to acceptors in the electrolyte (I_3_^−^ in Fig. 6b) and the capacitance of the system. Its lower value for photoelectrode C indicates in principle that thicker NTs presented higher surface area for electron transfer to the electrolyte. An inverse effect is observed in the generated photocurrent, a parameter that was larger for thicker NTs. This was likely due to the amount of dye absorbed on the surface. The best efficiency reached by the DSCs implementing the electrode C is 0.3% (c.f., Table 1). It is apparent that although the highest nanotube DSC efficiency is lower than the one obtained for the reference 800 nm PECVD film, the photocurrent measured is larger for the NT electrode. The observed tendency can be accounted for by the increment of the effective surface available in thicker nanotubes that should result in a higher dye load. This result is very significant since the nanotubes are almost four times thinner than the reference thin film. The increment of dye adsorbed might be related to the high specific surface area achieved with the nanotubes’ configuration and the smaller crystal size developed in these systems (Fig. 3). Further information on the charge transfer and electron transport processes of the cell can be obtained by Electrochemical Impedance Spectroscopy (EIS) and Intensity Modulated Photocurrent Spectroscopy (IMPS) (see also Section S1, Fig. S6 and S7 in the SI). The EIS spectra exhibited the typical shape of the current response of a DSC under small Fermi level perturbations, with a semicircle at intermediate frequencies attributed to the charge transfer between semiconductor and electrolyte (recombination reaction)^48^,^49^. The recombination resistance presents the usual exponential behaviour with respect to the applied bias^48^,^49^. The exponential behaviour of the experimental capacitance for the ZnO textured electrodes suggests that it corresponds to a chemical capacitance^50^. The lifetime of accumulated electrons is presented in Fig. S6)^51^. In comparison, a higher electron lifetime is observed for the reference DSC, a result that explains the larger open circuit voltage obtained for this electrode. The IMPS measurements provide useful information about the electron transport properties of the electrodes (Section S1 and Fig. S7). The calculated time constants (τ_IMPS_) are on the order of tens of milliseconds and present two different behaviors. An exponential dependence is found for the DSCs fabricated with the thinnest nanotubes (NT A), while for DSCs made with NTs B and C, τ_IMPS_ remains approximately constant. A similar constant behaviour is found for the reference thin film. Such a constant value of τ_IMPS_ can be related with the absence of an electron multiple trapping behavior in the ZnO electrode as previously reported^44^. Figure S6 presents the calculated values for the small-perturbation diffusion length L_n_, showing values around ten times larger than the ZnO wall thickness and values for the reference thin film cell one order of magnitude higher that those found for the nanotubes DSCs. A diffusion length much longer than the film thickness indicates that electron collection is quantitative, so this is not an issue for the performance of the cell. Joining the information provided by the EIS and IMPS analysis it is possible to approximately estimate the collection efficiency (Fig. S6). The graph shows clear differences between the efficiencies calculated for the different nanotubes devices, although in all the cases they converge to a maximum efficiency situation for low Fermi level energies (short-circuit conditions). Taking into account all this information we propose the mechanism in Fig. 6d) for the electron transport in 3D ZnO NTs. The nanotube wall is formed by ZnO globular columns (see Fig. 3) growing from the interface with the core. The photoelectrons are mainly generated in the outer shell of the ZnO wall and then migrate till the FTO substrate across the shell. The transport of electrons is effective within the column as such is the case in the columnar thin film and in good agreement with the single-crystalline condition (see Fig. 3e). However, while in the porous thin film configuration the columns are directly connected to the FTO electrode, in the NT architecture the path between the column where the photoelectron was generated and the electrode is more tortuous due to the grain boundaries between the columns and at their bases (i.e., in the inner face of the wall), involving as well the interface between the NT and the 200 nm ZnO blocking layer. This explains the shorter lifetimes and diffusion lengths and lower recombination resistances observed in NTs of increased thickness and when compared with the reference.

Despite possessing remarkable ZnO electron mobility, its known degradation by many usual dyes and instability in aqueous solution limits the overall cell performance and operation lifetime^52^. In an attempt to hinder its degradation, 1-D multishell based DSCs comprising ZnO NTs C, i.e. 250 nm in thickness, covered with a TiO_2_ anatase layer ca. 20 nm shell has been fabricated. However our first attempt has been discouraging as gathered in Table 1 since the preliminary results show a detrimental performance of the ZnO@anatase NTs in comparison with the single shell approach.

### 3D ZnO Nanotubes-Based Photonic Sensor

**Figure 7:**
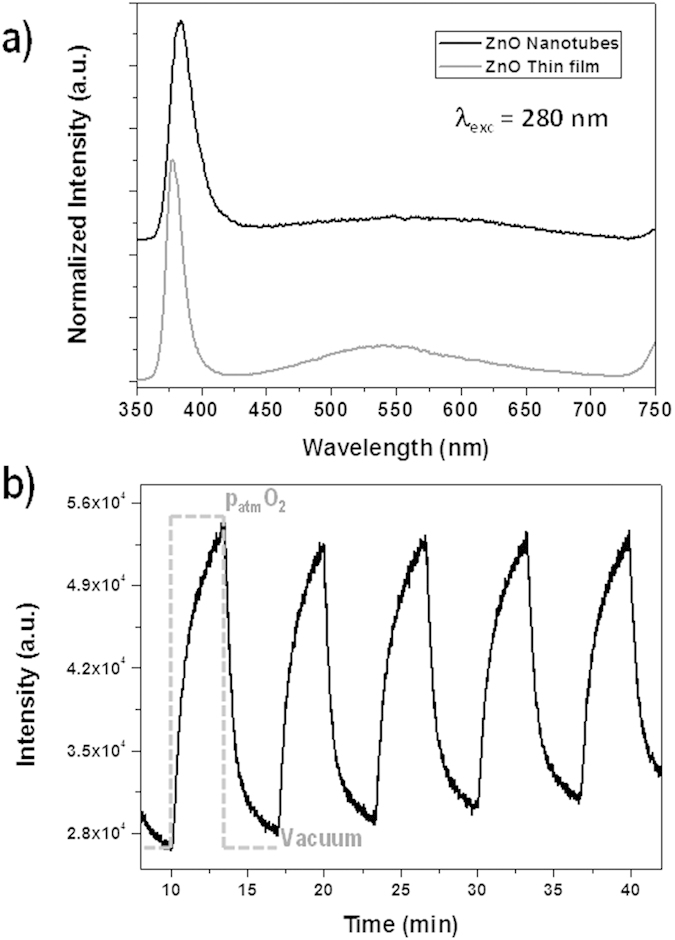
Luminescence oxygen sensing. (**a**) Room temperature luminescence emission spectra of the ZnO NTs deposited on fused silica compared with the poly-crystalline thin film reference deposited in the same experimental conditions. (**b**) Evolution of the ZnO NTs exciton photoluminescence intensity when successively exposed to cycles of vacuum and oxygen at atmospheric pressure. The experiment in each cycle was stopped when a 70% of the intensity change was measured before reaching the steady state. The excitation wavelength was 280 nm in both figures.

ZnO thin films and nanostructures have been widely applied as gas and volatile organic compounds sensors through both conductometric and optical approaches^11^,^26^,^53^,. Herein, we have aimed to the realization of an oxygen photonic sensor based on the UV emission quenching of the 3D ZnO NTs. Figure 7a shows the photoluminescence spectra of a reference nanocolumnar polycrystalline ZnO film and a 3D ZnO NTs sample. These two samples were deposited simultaneously on fused silica substrates, for the sake of comparison. The polycrystalline ZnO emission spectrum is dominated by a sharp and intense exciton ultraviolet (UV) emission at ~380 nm and a broad and low intense visible emission in the region 450–700 nm. The ZnO visible emission is attributed to defects as zinc vacancies (blue emission) and oxygen vacancies (green emission)^53^. In our case, the level of such defects is low, as addressed by the low intensity of the visible emission what have been attributed to a suppression of band-gap electronic defects by hydrogen species from the plasma during the synthesis. The photoluminescence spectra of the ZnO NTs samples show a defect related visible emission that is even of lower intensity than the reference film. This proves that the deposition on the 1D nanostructured substrates do not increase the number of defects in the resulting conformal oxide network. However, the PL spectra of both samples are not equivalent. Thus, while the PL band of the reference peak is sharper and centered at 378 nm, being originated by recombination of free excitons, the emission of ZnO nanotubes is broader and red-shifted, which indicates the domain of lower energy phonon replicas components^33^. These results are congruent with the higher stress, smaller crystallite size and lower texture development of the ZnO NTs sample with respect to the reference sample as discussed above. Moreover, a certain additional contribution to the observed red-shift of the UV PL band due to the anisotropic character of the oriented ZnO NTs emission cannot be discarded^54^. The excitonic emission of the ZnO NTs is a function of the oxygen concentration in the environment. Figure 7b plots the luminescent emission intensity determined at 384 nm during several cycles of vacuum and oxygen up to atmospheric pressure. The luminescence intensity decreases and increases reversibly with the partial oxygen pressure. This behaviour has been recently reported in thin films and was found to be associated to a high surface to bulk ratio. Furthermore, the nanostructuration of this material in the form of NTs enhances its properties, as we have just demonstrated with the fabrication of a photonic sensor, allowing its implementation in multifunctional devices.

## Conclusions

We have presented a reliable full vacuum methodology for the fabrication of semiconducting nanotubes made of ZnO and TiO_2_ with single and multishell configurations. The versatility of the plasma techniques such as PECVD for the growth of metal oxide layers has been exploited here for the formation of nanostructured 3D nanotubes with tailored shells in terms of microstructure, porosity, structure and thickness on an ample variety of substrates ranging from FTO supports to metal nanoparticles. The procedure provides hollow’s cross sections in the form of square or rectangle keeping memory of the flat surface of the organic single crystal used as templates. The performance of the 3D ZnO nanotubes as photoanode in a DSC has been analysed as a function of the shell thickness, finding an increase of efficiency with this parameter. In addition, the photoluminescence characterization has demonstrated that it is possible to synthetize high quality ZnO nanotubes with preferential emission in the UV range and low visible emission related to surface defects. The quenching of the luminescence in presence of gaseous oxygen has been applied to the fabrication of a reversible oxygen photonic sensor. Thus, we have demonstrated that it is possible to easily expand the sound knowledge in the fabrication of metal oxide thin films by PECVD and other related techniques^35^,^36^,^37^,^38^,^39^,^40^ to the formation of supported functional nanotubes with either domed or open configurations. The combination of the materials proposed herein with the deposition of metal layers opens a new route for the fabrication of single wire devices with applications in nanosensors, nanogenerators and photonics.

## Methods

### ONWs by PVD

Perylenediimide (2,9-dimethyl-anthra[2,1-def:6,5,10-d′e′f′]diisoquinoline-1,3,8,10-tetrone (Me-PTCDI) from Sensient Imaging Technologies, Octaethylporphyrin (OEP) and Nickel phthalocyanine (NiPc) from Aldrich were used as purchased. The PVD procedure for the supported formation of single crystal ONWs is fully described in previous refs 28, 29, 30, 31. It consist in the sublimation of organic molecules using a Knudsen cell in 0.02 mbar of Ar, at a growth rate about 0.3 Å/s with a controlled substrate temperature. The substrates temperatures were set at ~175 °C for the Me-PTCDI; ~130 °C for the OEP and ~230 °C for the NiPc.

### ZnO and TiO_2_ layers by PECVD

Both semiconducting oxides, ZnO and TiO_2_, were fabricated by PECVD in a microwave (2.45 GHz) ECR reactor with a down-stream configuration. The experimental setup for PECVD can be found elsewhere^34^,^38^. Diethylzinc (ZnEt2) and titanium tetraisopropoxide (TTIP) were utilized as corresponding precursors (Sigma Aldrich). Crystalline ZnO was grown at room temperature with oxygen as plasma gas. Total pressure in the chamber was settled at 1.5 × 10^−2^ mbar and plasma power at 400 W. TiO_2_-mesoporous was grown at the same conditions with a slightly higher pressure (8.6 × 10^−3 ^mbar). To get anatase crystalline phase, the substrate temperature was raised up to 250 °C. In the case of TiO_2_-nanoporous, argon and oxygen were used as plasma gases in a relation of 9:1, while keeping the total pressure close to 6 × 10^−3^ mbar and substrates at room temperature.

### Vacuum annealing

In order to remove the ONWs template, a heating treatment was applied to samples using a temperature of 350 °C at a pressure of 10^−6^ mbar for a period of 1 to 3 hours. No alteration of the vacuum level was detected during the process. A cold finger placed in the vacuum system was filled with liquid nitrogen to condensate the sublimated organic material. The composition of the ONWs working as template is an element of choice. In this case, we have preferentially worked with octaethyl porphyrin (OEP) and red perylene (MePTCDI) because of their low sublimation temperature (below 300 °C at 10^−2^ mbar) facilitates their evaporation through the inorganic walls porous microstructure. Results on metal phthalocyanine as ONWs are also included in the section regarding the emptying mechanism of the nanotubes in order to follow the metal in the molecule as trail of the evacuation pathway and in the real pictures of the solar cells devices shown in Fig. 1.

### Characterization

SEM micrographs were acquired in a Hitachi S4800 working at 2 kV. The samples were dispersed onto Holey carbon films on Cu or Ni grids from Agar scientific for TEM characterization. EDX maps were acquired with a FEI Tecnai Orisis TEM/STEM working at 200 kV and equipped with a Super-X EDX system. EDX data were post-processed with the open source Hyperspy software (www.hyperspy.org), in order to obtain more quantitative compositional maps of the multishell system. HAADF-STEM and HRTEM studies were carried out with both Osiris and FEI Tecnai F30 S-Twin STEM microscopes operating at 200 kV. HR-STEM images were acquired with a probe-corrected FEI TITAN3 80-300 microscope operating at 300 kV. The crystal structure was analyzed by XRD in a Siemens D5000 spectrometer operated in the θ–2θ configuration and using the Cu Kα (1.5418 Å) radiation as an excitation source. UV-Vis analysis of the samples was done in a Cary 100 spectrometer from Varian. Fluorescence spectra were recorded in a Jobin Yvon Fluorolog-3 spectrofluorometer using an excitation wavelength of 280 nm and scanning the emission spectra between 350 and 750 nm with a 2 nm monocromator step.

## Additional Information

**How to cite this article**: Filippin, A. N. *et al*. Vacuum template synthesis of multifunctional nanotubes with tailored nanostructured walls. *Sci. Rep.* **6**, 20637; doi: 10.1038/srep20637 (2016).

## Supplementary Material

Supplementary Information

Supplementary Video S1

Supplementary Video S2

## Figures and Tables

**Table 1:** Photovoltaic parameters for ZnO-based DSCs as a function of the wall thickness.

	J_sc_(mA/cm^2^)	V_oc_(mV)	FF(%)	η(%)
NT A	0.39 ± 0.21	610 ± 25	40 ± 1	0.10 ± 0.05
NT B	0.73 ± 0.15	500 ± 30	44 ± 2	0.20 ± 0.05
NT C	1.48 ± 0.25	460 ± 20	50 ± 1	0.30 ± 0.05
Reference	1.05 ± 0.30	670 ± 15	45 ± 1	0.50 ± 0.1
NT C@anatase	0.99 ± 0.13	588 ± 8	43.4 ± 5.3	0.20 ± 0.05

- Mean photovoltaic parameters values and estimated errors have been obtained from data of three devices with the same configuration. A device fabricated with a 800 nm thin film is used as reference.
