# Supplementary material for: Vacuum template synthesis of multifunctional nanotubes with tailored nanostructured walls
Source: Sci Rep. 2016 Feb 10;6:20637. doi: 10.1038/srep20637 (PMC4748298; doi:10.1038/srep20637)
Supplement: Supplementary Information [file srep20637-s1.pdf]

Supporting Information

Vacuum template synthesis of multifunctional nanotubes with tailored nanostructured walls

A. Nicolas Filippin, Manuel Macias-Montero, Zineb Saghi, Jesús Idígoras, Pierre Burdet, Angel Barranco, Paul Midgley, Juan A. Anta and Ana Borras

We have presented a reliable full vacuum methodology for the fabrication of semiconducting nanotubes made of ZnO and TiO_2 with single and multishell configurations. The versatility of the plasma techniques such as PECVD for the growth of metal oxide layers has been exploited here for the formation of nanostructured 3D nanotubes with tailored shells in terms of microstructure, porosity, structure and thickness on an ample variety of substrates ranging from FTO supports to metal nanoparticles. The procedure provides hollow’s cross sections in the form of square or rectangle keeping memory of the flat surface of the organic single crystal used as templates. The performance of the 3D ZnO nanotubes as photoanode in a DSC has been analyzed as a function of the shell thickness, finding an increase of efficiency with this parameter. In addition, the photoluminescence characterization has demonstrated that it is possible to synthetize high quality ZnO nanotubes with preferential emission in the UV range and low visible emission related to surface defects. The quenching of the luminescence in presence of gaseous oxygen has been applied to the fabrication of a reversible oxygen photonic sensor. Thus, we have demonstrated that it is possible to easily expand the sound knowledge in the fabrication of metal oxide thin films by PECVD and other related techniques to the formation of supported functional nanotubes with either domed or open configurations. The combination of the materials proposed herein with the deposition of metal layers opens a new route for the fabrication of single wire devices with applications in nanosensors, nanogenerators and photonics.

Methods. *Solar cells.* Counter electrode: FTO substrates of 2.5 x 2 cm provided by XOP Glass were drilled in two points for later electrolyte injection, rinsed with acetone, isopropanol and absolute ethanol and heated to 500 ºC for 1 hour. 12 μL of plastisol (Solaronix) are dispersed on the substrates, dried in air and heated in a furnace for 20' at 400 ºC.

Electrodes: FTO substrates were cleaned just as the counter electrodes. An active area of 0.7 cm^2 was defined with an aluminum foil mask and a blocking layer of 200 nm of ZnO was deposited by PECVD. This ZnO acts as a hole blocking layer and provides the necessary roughness for the growth of ONWs. ZnO 3D NTs with different thicknesses were fabricated by PECVD onto FTO electrode through a mask to delimitate a covered area of 7 x 10 mm^2. Samples were heated up to 80ºC before immersing in the dye solution (0.5 mM solution of N719 dye (Solaronix)) to prevent adsorption of air moisture. To avoid dye aggregation and NTs deterioration, the electrodes were immersed in the dye solution for 1 hour. Afterwards they were rinsed with an ethanol baker and dried under a flow of nitrogen. A similar procedure was carried out for the fabrication of the ZnO@anatase nanowires. In this case an additional step was applied for the formation of anatase on the as-grown of the ZnO nanowires. Substrate temperature was increased up to 250 ºC and the plasma reactor was feeded with TTIP precursor as detailed in the main text.

The electrolytic solution was prepared by addition of 0.6m 1, 2-dimethyl-3-propylimidazole iodine (DMPII), 0.1m LiI, 0.5m 4-tertbutyl-pyridine (TBP), 0.05m I2 and 0.1m guanidinium thiocyanate (GuSCN) to a mixture of acetonitrile/valeronitrile (85/15).

DSC: The solar-cell devices were characterized using a solar simulator with an AM1.5G filter (ABET). A reference solar cell with temperature output (Oriel, 91150) was used

for calibration. EIS measurements were performed under light with perturbations in the 10^{5}–10^{-3} Hz range and the IMPS measurements in the 10^{5}–10^{-3} Hz range. For all the small perturbation techniques, we used an LED LUXEON collimated (540 nm) source and an Autolab/PGSTAT302N potentiostat. Zview equivalent circuit modelling software (Scribner) was used to fit the EIS data, including the distributed element DX11 (transmission line model). The NOVA 1.7 software was used to analyze the IMPS data.

Oxygen Gas Sensor. A thin layer of columnar SiO_2 (300 nm) was deposited by glancing angle deposition (GLAD) as described elsewhere.[36] ONWs of MePTCDI were then grown by PVD, which were ultimately covered by a 200 nm ZnO shell and emptied after annealing in vacuum at 350 ºC for 3 hours. Reference thin film sensors were also fabricated using the same PECVD conditions.

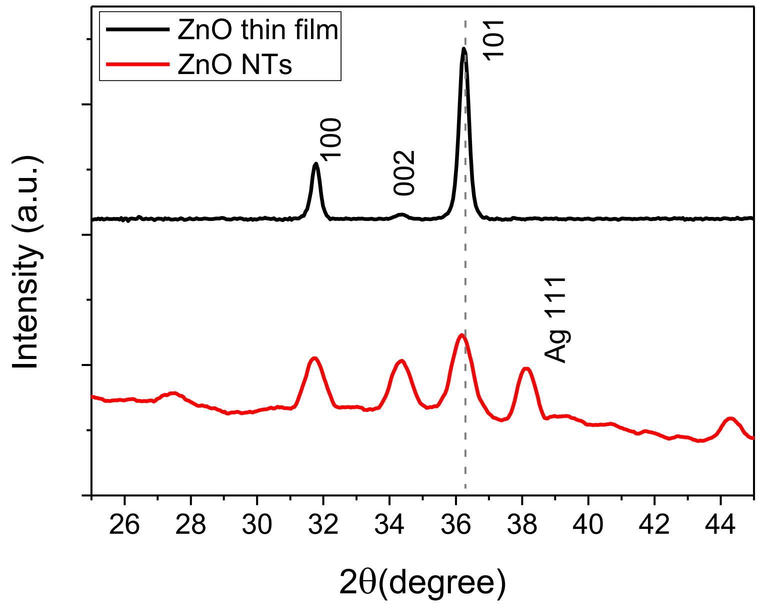

Figure S1. XRD spectra of ZnO thin films and nanotubes.

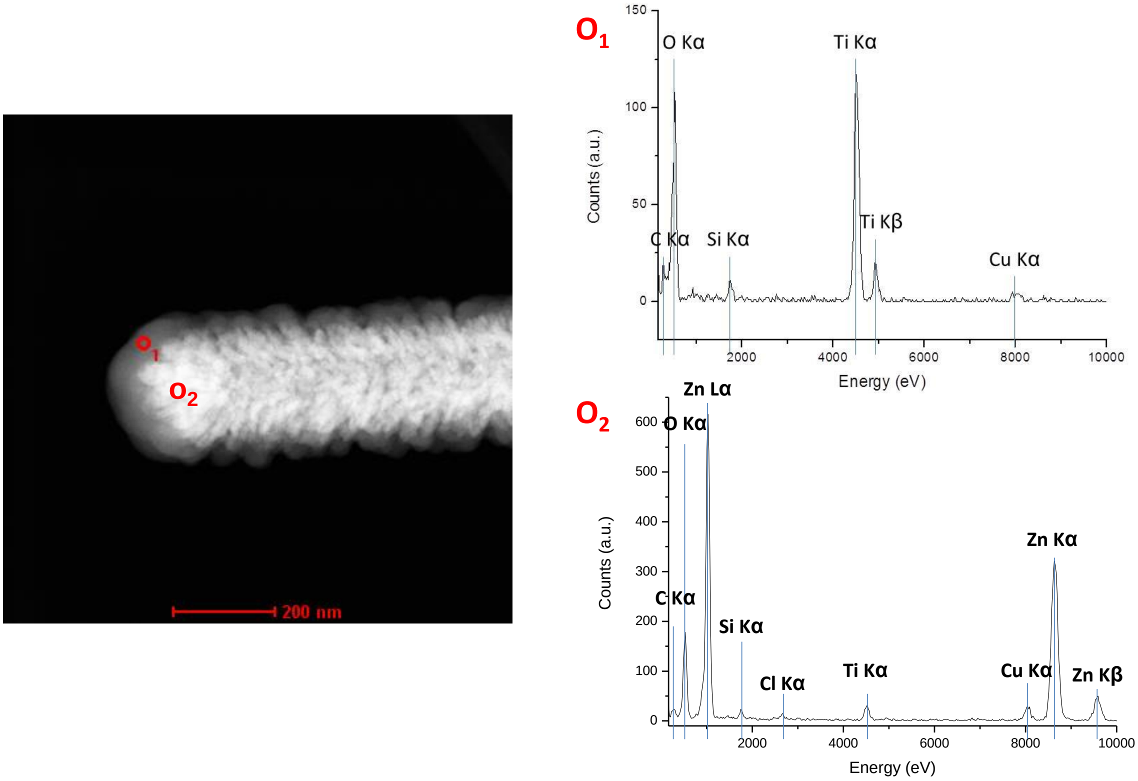

Figure S2. EDX (right) spectra of the selected points in the HAADF-STEM micrograph on the left) showing the different composition of the shells in good agreement with the sequential deposition of ZnO (inner shell) and TiO_2 (outer shell).

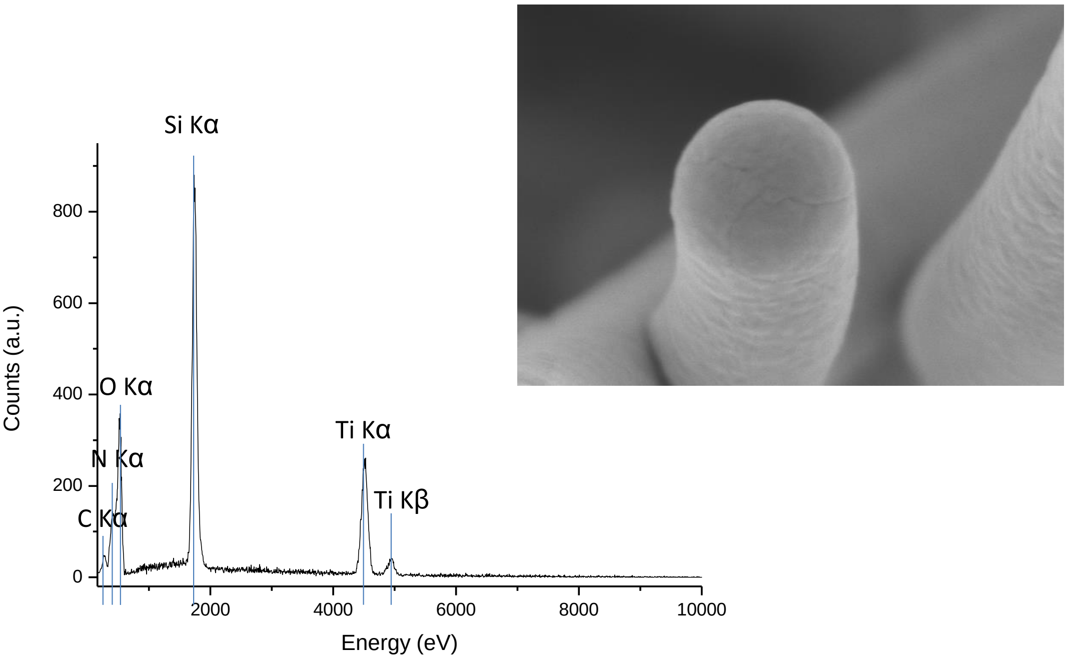

Figure S3. EDX spectra of the selected nano-TiO_2 NT formed after evacuation of the NiPc@nano-TiO_2 hybrid nanowire showing no trace of Ni after the annealing treatment.

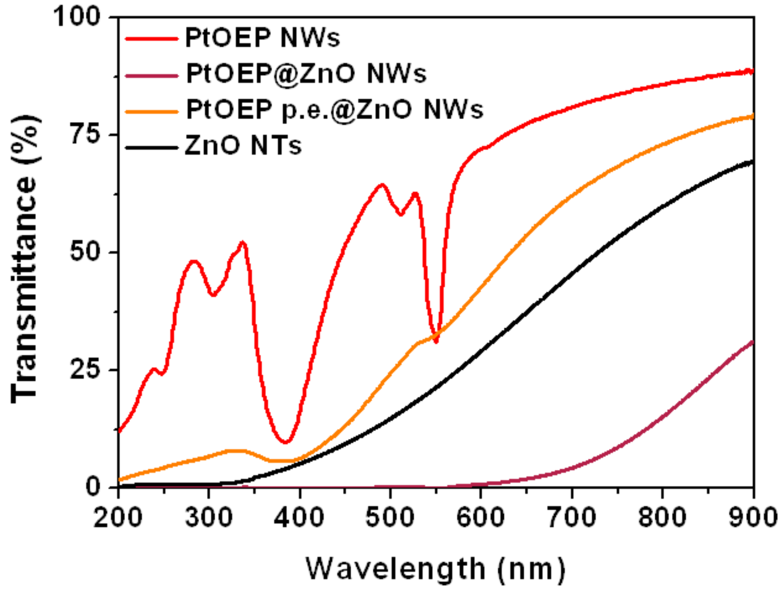

Figure S4. UV-Vis spectra of PtOEP NWs, PtOEP@ZnO NWs (hybrid nanowires), PtOEP@ZnO partially evacuated (p.e.) NWs (30 minutes of annealing) and ZnO nanotubes (fully evacuated, annealing during 60 minutes) on a fused silica substrate previously coated with a GLAD-SiO_2 thin film. The annealing treatment for evacuation of the organic template was carried out at 280 ºC and in high vacuum conditions (10^{-6} mbar). In all the cases the spectra are significantly dominated by light scattering effects related with the size and distribution of the 1D supported nanostructures.

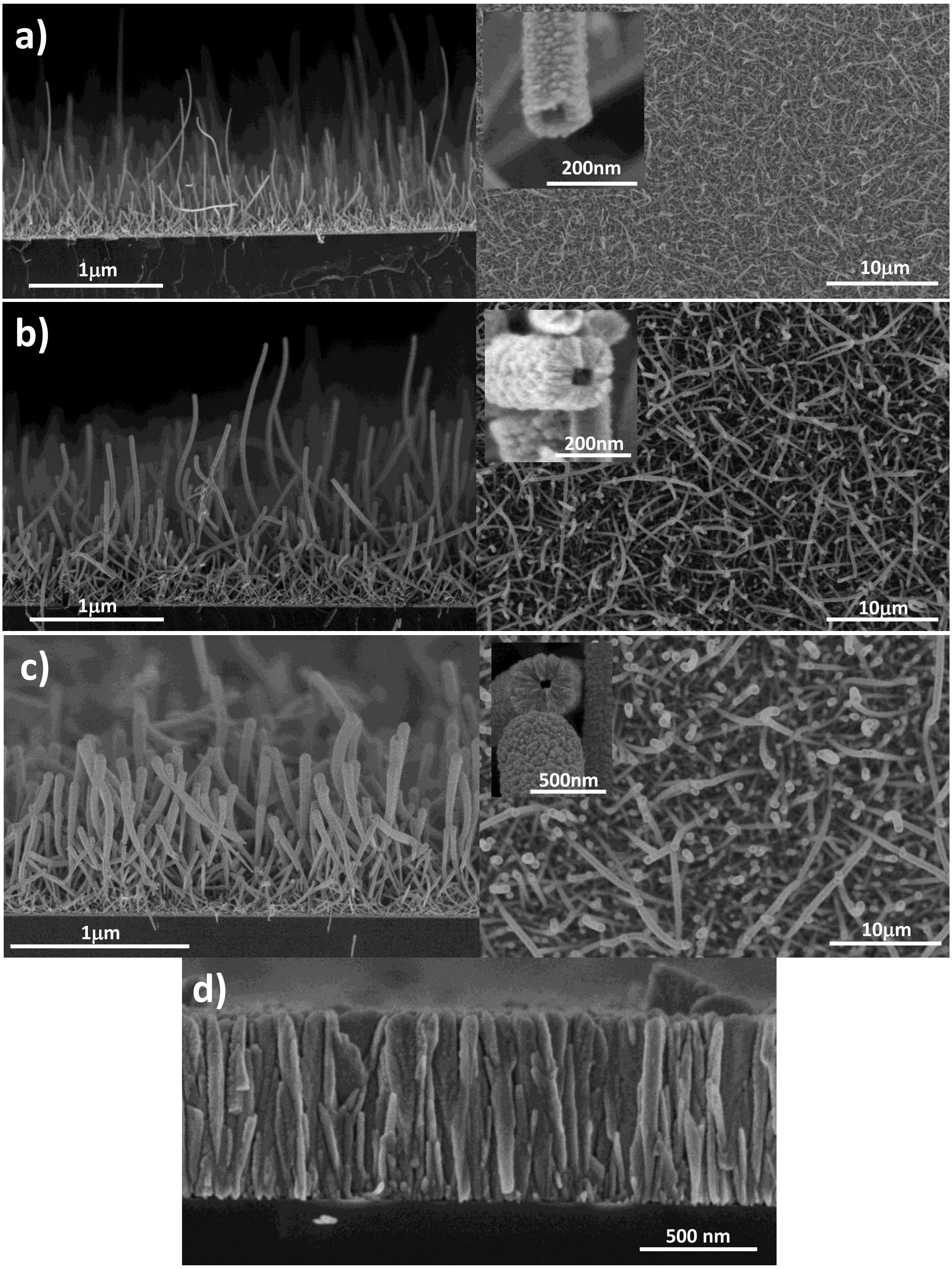

Figure S5. SEM of ZnO nanotubes and porous thin film electrodes for DSC. a-c) Cross section (left) and normal (right) view SEM micrographs of ZnO nanotubes electrodes implemented in DSCs. Insets show magnified views of single nanotubes to show the increment in the thickness of the walls. d) Cross- section SEM micrograph of the ZnO thin film electrode.

Section S1. Charge transfer and Electron transport properties obtained by Electrochemical Impedance Spectroscopy (EIS) and Intensity Modulated Photocurrent Spectroscopy (IMPS)

Further information on the electron transport properties can be obtained by Electrochemical Impedance Spectroscopy (EIS) and Intensity Modulated Photocurrent Spectroscopy (IMPS). The EIS spectra exhibited the typical shape of the current response of a DSC under small Fermi level perturbations, with a semicircle at intermediate frequencies attributed to the charge transfer between semiconductor and electrolyte (recombination reaction).[1] Fitting these spectra by using the transmission line model accounting for transport and recombination in the semiconductor electrode provides specific data for the recombination resistances and the capacitance of the system as a function of the applied bias. The variation of these parameters as a function of the Fermi level voltage is presented in Figure S6 a-c). The recombination resistance measured for the nanotubes devices presents low values, especially in the NT C case, compared with the reference DSC. This could be attributed to the worse grain connectivity of the nanotubes compared with the thin film that would give rise to higher electron recombination losses. This tendency can be correlated with the low open circuit voltage measured for thick nanotubes (i.e. NT C condition). It is also worth noting that for thin nanotubes (NT A) the behavior is quite similar to the reference. The recombination resistance presents an exponential behavior with respect to the applied bias that can be accounted for by the equation:[2]

$$R_{rec} = R_{rec,0} \exp\left(\frac{-\beta(E_F - E_F^0)}{k_B T}\right) \qquad \text{Eq. S1}$$

where k_B is the Boltzmann constant, T the absolute temperature and E_F, E_F^0 are the electron Fermi levels in the semiconductor under bias and in the dark, respectively. As shown in Fig. S6 b) and predicted by Eq. S1, the recombination resistance decreases with the bias potential, according to $-eV = E_F - E_F^0$. This tendency means that the Fermi level is raised towards the conduction band as a higher number of electrons are accumulated in the photoanode. The slope of the recombination resistance with respect to the applied potential depends on the adimensional parameter β, which is related to the non-ideality of the recombination reaction.[2] The experimental data, including those corresponding to the reference electrode, fit well to Eq. 1 with β~0.4. This low β value should be taken as a factor contributing to lower the efficiency of our devices.

The capacitance data can be fitted to the theoretical equation:[3, 4]

$$C_\mu = C_{\mu,0} \exp\left(\frac{\alpha(E_F - E_F^0)}{k_B T}\right) \qquad \text{Eq. S2}$$

where α is an adimensional parameter that describes the average energy of an exponential distribution of trapping states situated below the semiconductor conduction band. The

exponential behavior of the experimental capacitance for the two ZnO textured electrodes suggests that it corresponds to a chemical capacitance,[4] as described by Eq. S2. However, the extracted values of α are on the order of 0.05, below the typical values of ZnO DSCs found in bibliography. The differences appreciated in the absolute values of the capacitance (Fig. S6 c), particularly the high values found for NT B and C, can be attributed to the increment of the wall thickness. The lifetime of accumulated electrons in the semiconductor electrode have been obtained via the equation:[5]

$$\tau_n(n) = \left(\frac{\delta J_R}{\delta n}\right)^{-1} = R_{rec} \cdot C_\mu \qquad \text{Eq. S3}$$

Experimental lifetimes extracted from the EIS fittings are presented in Fig. S6 (d). Comparing the data for the same Fermi level, the obtained values depict a decrease of the lifetime when incrementing the NT wall thickness and suggest a high recombination probability in agreement with the lower open circuit voltage observed in these latter cases. This might be due to the increment in the density of grain boundaries along the nanotubes where the electronic transport would be hindered. In comparison, a higher electron lifetime is observed for the reference DSC, a result suggesting a more efficient electron transport through the film than through the ZnO nanotubes. The Intensity Modulated Photocurrent Spectroscopy measurements provide complementary information about the electron lifetime and electron-hole recombination. The IMPS data correspond to values of the transfer function (H = H'+iH'') between the modulated light intensity and the measured AC current of the cell at short-circuit. The representation of H'' versus the frequency in Fig. S7 (a)-(c) provides accurate information of the time constant derived from the IMPS. The time constant τ_{IMPS} is obtained as the inverse of the frequency (ω_{max}) at which the imaginary component (H'') is maximum. The calculated time constants are on the order of tens of milliseconds. There are two different behaviors when representing τ_{IMPS} vs. Fermi level. An exponential dependence is found for the DSCs fabricated with the thinnest nanotubes (NT A), while for DSCs made with NTs B and C, τ_{IMPS} remains constant. A similar constant behavior is found for the reference thin film. Such a constant value of τ_{IMPS} can be related with the absence of electron multiple trapping in the ZnO electrode where it would be observed above a certain thickness threshold. The diffusion length, defined as the average distance that electrons can travel in the photoanode before recombination, provides information about charge collection processes in photoanodes.[2] For small perturbations of the Fermi level, a small-perturbation diffusion length L_n is defined. This parameter can be obtained from EIS measurements by extracting the recombination and the transport resistances.[6]. However, it has to be noted that extraction of the transport resistance is not very accurate in our case, since the numerical fitting gives large numerical errors for this parameter when it is of the same order of magnitude that the counter-electrode resistance.[7]. Furthermore, the 45o-straight line feature appearing at high frequencies, an indication of electron transport in nanostructured metal-oxide electrodes,[8], does not appear clearly in ZnO-based electrodes.[9] As mentioned in the main text, despite that, this method provides a valuable estimation on the order of magnitude of the

electron diffusion length. Fig. S6 e) presents the calculated values for this magnitude for the different ZnO nanotubes based DSCs under consideration, showing values around ten times larger than the ZnO wall thickness. However, the values calculated for the reference thin film cell are one order of magnitude higher that that found for the nanotubes DSCs. In any case the values obtained are large enough to not hamper the performance of the solar cells. Joining the information provided by the EIS and IMPS analysis it is possible to calculate the collection efficiency as 1 - τ_{IMPS} / τ_n. Due to the small ZnO thicknesses involved in this work, we expect this equation to be reasonably valid with an acceptable level of inaccuracy.[10] Given the possible deviations of the calculated collection efficiencies from real ones, for values < 0.9, focus is made on the trends, not on the absolute values.[11] Fig. S6 f) gathers the obtained values plotted as a function of the position of the Fermi level. The graph shows clear differences between the efficiencies calculated for the different nanotubes devices, although in all the cases they converge to a maximum efficiency situation for low Fermi level energies.

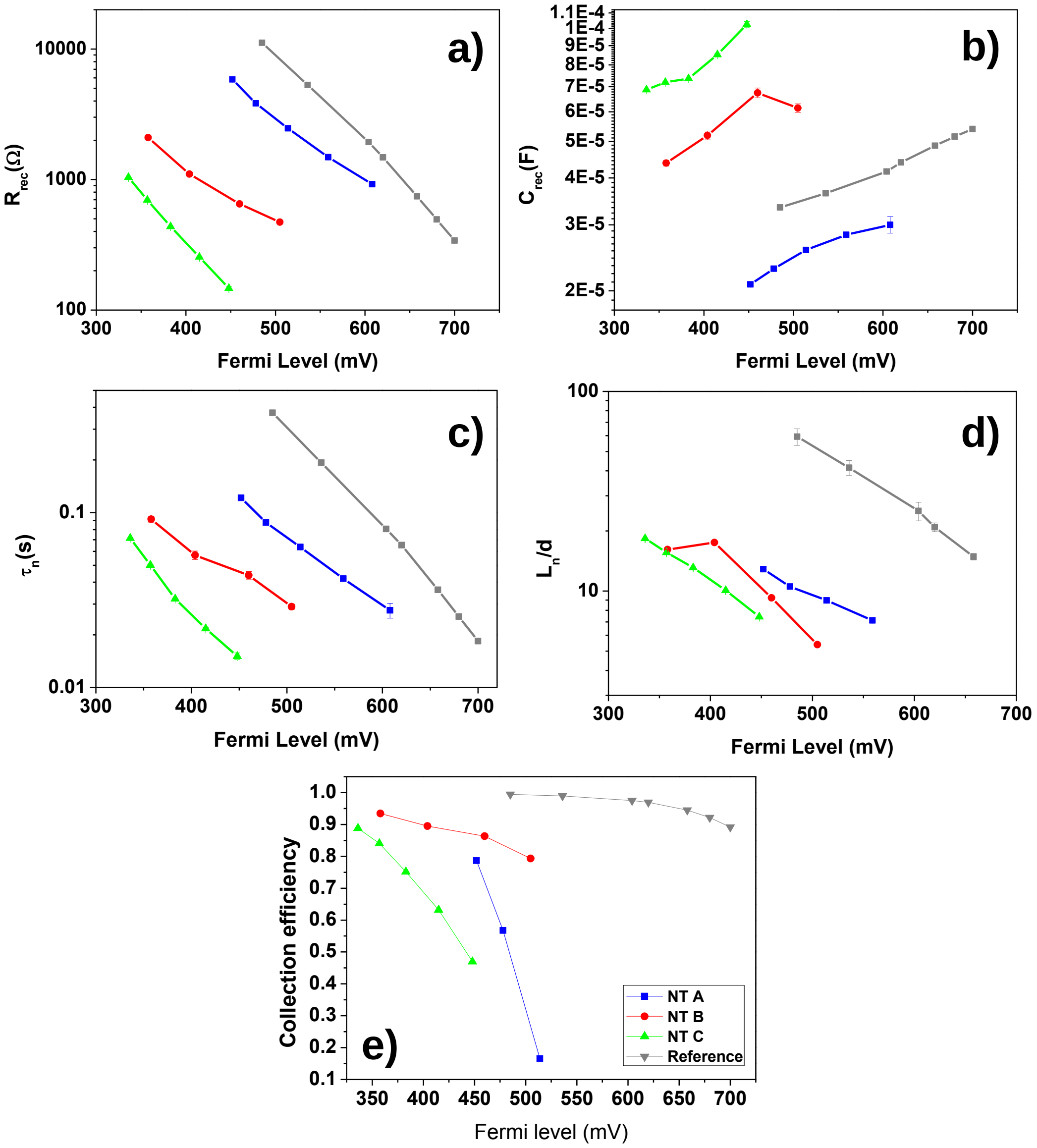

Figure S6. a-c) Impedance spectroscopy results of DSCs fabricated with ZnO nanotubes. Impedance parameters extracted from the fitting of the EIS spectra at various applied potentials: (a) Recombination resistance, (b) Capacitance, (c) Lifetime. Collection efficiency: d) electron diffusion length, estimated as the ratio of recombination and transport resistances, e) Collection efficiency calculated as 1 - τ_{IMPS} /τ_n.

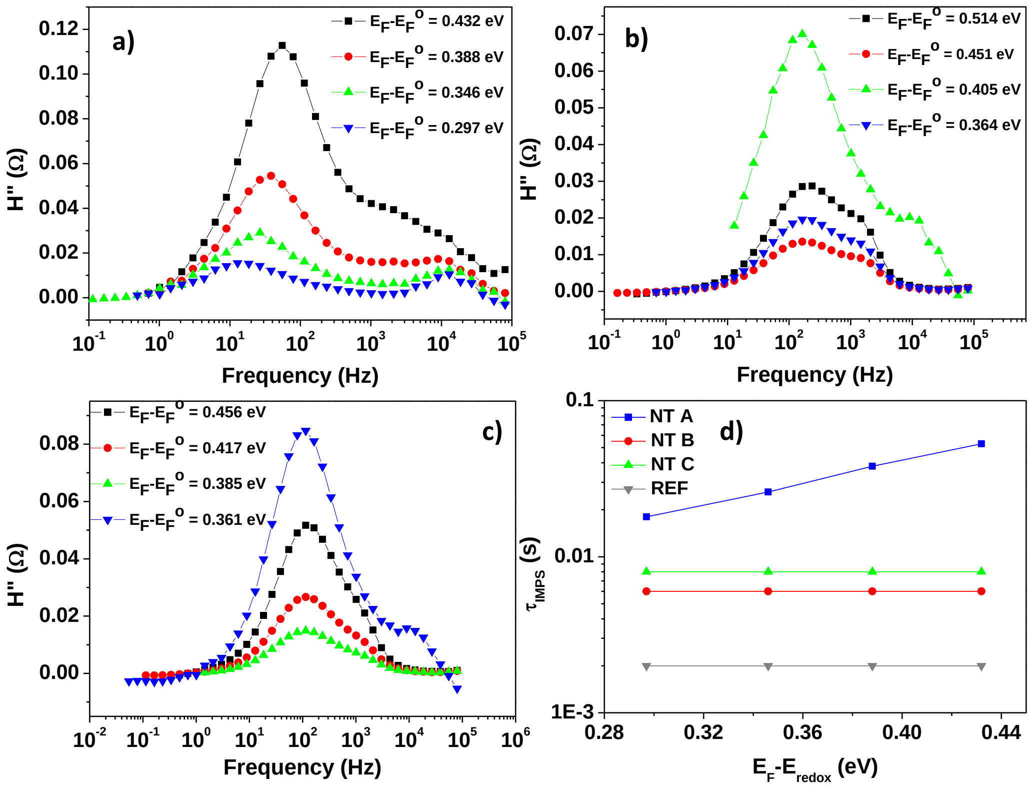

Figure S7. IMPS of DSCs implementing ZnO nanotubes. - (a)-(c) Imaginary IMPS component plotted versus the frequency at different light intensities for NTs A, B and C devices. (d) IMPS plotted versus the Fermi level for the different DSCs, including the ZnO porous thin film device as a reference.

References

[1] a) E. Guillen, L. M. Peter, J. A. Anta. *J. Phys. Chem. C,* 2011, **115**, 22622. b) F. Fabregat-Santiago, J. Bisquert, G. Garcia-Belmonte, G. Boschlo, A. Hagfeldt., *Solar Ener. Mater. Solar Cells*, 2005, **87**, 117. c) F. Fabregat-Santiago, G. Garcia-Belmonte, I. Mora-Sero, J. Bisquert., *Phys. Chem. Chem. Phys*,. 2011, **13**, 9083.
[2] J. Bisquert, I. Mora-Sero. *J. Phys. Chem. Letters* **2010**, *1*, 450-456.
[3] R. Mohammadpour, A. Irajizad, A. Hagfeldt, G. Boschloo., *ChemPhysChem*, 2010, **11**, 2140.
[4] J. Bisquert., *Phys. Chem. Chem. Phys.*, 2003, **5**, 5360.
[5] J. Bisquert, F. Fabregat-Santiago, I. Mora-Sero, G. Garcia-Belmonte, S. Gimenez. J. *Phys. Chem. C*, 2009, **113**, 17278.
[6] Q. Wang, S. Ito, M. Gratzel, F. Fabregat-Santiago, I. Mora-Sero, J. Bisquert, T. Bessho, H. Imai., *J. Phys. Chem. B*, 2006, **110**, 25210.
[7] H. Wang, L.M. Peter., *J. Phys. Chem. C*, 2009, **113**, 18125.
[8] J. Bisquert., *J. Phys. Chem. B*, 2002, **106**, 325.
[9] J. A. Anta, E. Guillen, R. Tena-Zaera., *J. Phys. Chem. C,* 2012, **116**, 11413.
[10] L. Bertoluzzi, S. Ma. *Phys. Chem. Chem. Phys.,* 2013, **15**, 4283.
[11] V. Sivaram, J. Kirkpatrick, H. Snaith., *J. Appl. Phys.,* 2013, **113**, 063709.
